# Antimicrobial susceptibility of commensal *Neisseria* in a general population and men who have sex with men in Belgium

**DOI:** 10.1038/s41598-021-03995-1

**Published:** 2022-01-07

**Authors:** Jolein Gyonne Elise Laumen, Christophe Van Dijck, Saïd Abdellati, Irith De Baetselier, Gabriela Serrano, Sheeba Santhini Manoharan-Basil, Emmanuel Bottieau, Delphine Martiny, Chris Kenyon

**Affiliations:** 1 Department of Clinical Sciences, Institute of Tropical Medicine Antwerp, Nationalestraat 155, 2000 Antwerp, Belgium; 2 Laboratory of Medical Microbiology, University of Antwerp, Wilrijk, Belgium; 3 Department of Microbiology, Laboratoire Hospitalier Universitaire de Bruxelles, Pôle Hospitalier Universitaire de Bruxelles, Université Libre de Bruxelles, Brussels, Belgium; 4 Faculté de Médecine et Pharmacie, Université de Mons, Mons, Belgium; 5 Department of Medicine, University of Cape Town, Cape Town, South Africa

**Keywords:** Antimicrobial resistance, Clinical microbiology

## Abstract

Non-pathogenic *Neisseria* are a reservoir of antimicrobial resistance genes for pathogenic *Neisseria meningitidis* and *Neisseria gonorrhoeae*. Men who have sex with men (MSM) are at risk of co-colonization with resistant non-pathogenic and pathogenic *Neisseria*. We assessed if the antimicrobial susceptibility of non-pathogenic *Neisseria* among MSM differs from a general population and if antimicrobial exposure impacts susceptibility. We recruited 96 participants at our center in Belgium: 32 employees, 32 MSM who did not use antibiotics in the previous 6 months, and 32 MSM who did. Oropharyngeal *Neisseria* were cultured and identified with MALDI-TOF–MS. Minimum inhibitory concentrations for azithromycin, ceftriaxone and ciprofloxacin were determined using E-tests^®^ and compared between groups with non-parametric tests. Non-pathogenic *Neisseria* from employees as well as MSM were remarkably resistant. Those from MSM were significantly less susceptible than employees to azithromycin and ciprofloxacin (*p* < 0.0001, *p* < 0.001), but not ceftriaxone (*p* = 0.3). Susceptibility did not differ significantly according to recent antimicrobial exposure in MSM. Surveilling antimicrobial susceptibility of non-pathogenic *Neisseria* may be a sensitive way to assess impact of antimicrobial exposure in a population. The high levels of antimicrobial resistance in this survey indicate that novel resistance determinants may be readily available for future transfer from non-pathogenic to pathogenic *Neisseria*.

## Introduction

*Neisseria gonorrhoeae* and *N. meningitidis* are becoming increasingly resistant to antimicrobials. For *N. gonorrhoeae* this concerns last-resort antimicrobials such as ceftriaxone and azithromycin^1,2^. Numerous studies have documented that for both species, much of this resistance has been acquired from the non-pathogenic *Neisseria* species that are a key component of a healthy oropharyngeal microbiome^3–8^. The most prominent genes involved in this transformation include *penA, mtrCDE, rplB, rplD, rplV, parC,* and *gyrA*. The acquisition of sections of these genes from non-pathogenic *Neisseria* has played an important role in the acquisition of penicillin, cephalosporin, macrolide, and/or fluoroquinolone resistance in *N. meningitidis* and *N. gonorrhoeae*^5,9,10^. Recent studies have established that uptake of DNA from non-pathogenic *Neisseria* was responsible for the majority of fluoroquinolone resistance in *N. meningitidis* and most azithromycin resistance in *N. gonorrhoeae* in Germany and the United States^4,7,11^. Non-pathogenic *Neisseria* have therefore gained interest as “canaries in the coalmine” for potential future resistance development in pathogenic *Neisseria*^9,12,13^.

Despite their importance as reservoirs of antimicrobial resistance (AMR), very few studies have explored the antimicrobial susceptibilities of contemporary non-pathogenic *Neisseria*. Studies of historical isolates found that non-pathogenic *Neisseria* were generally less susceptible to antimicrobials than pathogenic *Neisseria*^9,13^. In the last decade, however, few surveys have reported data on antimicrobial susceptibility of non-pathogenic *Neisseria* isolates. Two studies reported high minimum inhibitory concentrations (MICs) for macrolides, cephalosporins and fluoroquinolones among *N. lactamica* isolates from children in Japan and China in 2015^14,15^. One study found 93% fluoroquinolone resistance among commensal *Neisseria* from asymptomatic *N. meningitidis* carriers in China^7^. Two other studies were surveys among men who have sex with men (MSM) visiting a sexual health clinic in Vietnam in 2016 and Belgium in 2019^8,16,17^. Both reported reduced susceptibility of non-pathogenic *Neisseria* to the antimicrobials currently used to treat gonorrhoea—azithromycin, and ceftriaxone. The high azithromycin and ceftriaxone MICs of non-pathogenic *Neisseria* among MSM is of particular concern as gonococcal AMR has frequently emerged in MSM^18–20^. MSM are also often co-colonised by *N. meningitidis* and *N. gonorrhoeae* in their pharynx^21–26^.

Beyond these studies, very little is known about the epidemiology of antimicrobial susceptibilities in non-pathogenic *Neisseria.* In particular, little is known about their susceptibility in contemporary general adult populations.

It is not even known if the non-pathogenic *Neisseria* are more or less resistant in MSM than the general population and how MICs vary in relation to recent antimicrobial consumption.

Therefore, the aim of the current study was to compare the antimicrobial susceptibility of oropharyngeal *Neisseria* between MSM who recently used antimicrobials, MSM who did not, and employees of our institute as representatives of the general population in Belgium.

## Methods

### Survey population

This cross-sectional survey included 64 MSM and 32 employees.

The 64 MSM participated in a single centre randomized clinical trial (PReGo) at the Institute of Tropical Medicine (ITM) in Antwerp, Belgium in 2019–2020. PReGo was a placebo-controlled trial that assessed the efficacy of an antiseptic mouthwash (Listerine™) to prevent STIs among 343 MSM^27^. Taking HIV pre-exposure prophylaxis (PrEP) and having a history of gonorrhoea, chlamydia or syphilis in the previous two years was an inclusion criterium of that study. For the current survey, MSM were sampled at their first study visit, before administration of the PReGo study mouthwash. PReGo participants were enrolled into two groups, depending on their history of antimicrobial exposure.

#### Group I: MSM who recently used antimicrobials (n = 32)

The first 32 PReGo participants who used at least one antimicrobial in the previous 6 months were included in this group.

#### Group II: MSM who did not recently use antimicrobials (n = 32)

The first 32 PReGo participants who did not use any antimicrobial in the previous 6 months were included in this group.

#### Group III: Representatives of the general population: ITM employees who did not recently use antimicrobials (n = 32)

In June 2020, ITM employees were invited to participate by posters and by word of mouth. Candidates who used an antimicrobial in the previous 6 months were excluded. The first 32 eligible employees (male or female) presenting to the study team were included in this survey.

### Data collection and sampling procedure

All participants provided written informed consent prior to the collection of data and samples. Baseline characteristics were noted (including self-reported age, sex, antimicrobial use in the previous 6 months). Oropharyngeal samples were taken by a study physician who rubbed both tonsillar pillars and the posterior oropharynx with an ESwab™ (COPAN Diagnostics Inc., Italy).

### Sample processing

#### Culture and identification of Neisseria species

ESwabs™ were inoculated onto Columbia Blood Agar and Modified Thayer-Martin Agar using the streak plate technique and incubated at 35–37 °C and 5% carbon dioxide. Plates were examined after 48 h and Gram negative, oxidase positive colonies were selected, enriched and stored in Skim-milk at − 80 °C.

Isolates were identified to the species level using Matrix-Assisted Laser Desorption/Ionization-Time-of-Flight mass spectrometry (MALDI-TOF MS), on a MALDI Biotyper^®^ Sirius IVD system using the MBT Compass IVD software and library (Bruker Daltonics, Bremen, Germany). Briefly, each bacterial isolate was smeared twice on a polished steel target plate and then covered with 1 μL of α-cyano-4-hydroxycinnamic acid (CHCA) matrix solution. After drying, the target plate was loaded into the instrument. The spectra were acquired in linear mode in a mass range of 2–20 kDa and subsequently compared to the library that included 9607 spectra at that time. Identification results were classified as reliable or unreliable according to recommended cut-off values of 1.7 and 2 for validated results for the genus and species levels, respectively. Only isolates belonging to the genus *Neisseria* were included in further analyses. Isolates identified as *N. macacae* were grouped into one category with *N. mucosa,* whereas isolates identified as *N. perflava* and *N. flavescens* were grouped into one category with *N. subflava*^28^.

#### Antimicrobial susceptibility determination

Minimum inhibitory concentrations (MICs) of *Neisseria* species to azithromycin, ceftriaxone, and ciprofloxacin were determined on GC agar plates using ETEST^®^ (bioMérieux Marcy-l'Étoile, France) incubated for 24 h at 36.5 °C and 5–7% CO_2_, and expressed in mg/L. Lack of bacterial growth during susceptibility testing resulted in missing values for that isolate.

### Statistics

#### Neisseria prevalence

Prevalence was expressed as the proportion of participants from whom a certain species was isolated. Prevalence was compared between groups using Chi square tests.

#### Neisseria species richness

*Neisseria* species richness was defined as the number of different non-pathogenic *Neisseria* species per participant. Species richness was reported as median (interquartile range) and compared between groups using Kruskal–Wallis rank sum tests. If no significant differences were observed between the two groups of MSM, their data were combined.

#### Antimicrobial susceptibility

To enable statistical testing, MICs above the maximum or below the minimum level of the ETEST strip were simplified as follows: azithromycin MIC > 256 mg/L was recoded as 512 mg/L; ceftriaxone MIC < 0.016 mg/L as 0.008 mg/L; and ciprofloxacin MIC > 32 mg/L as 64 mg/L. If multiple colonies of the same species were isolated from the same participant, we calculated the median MIC for that species per participant. MICs were reported as median (interquartile range) and compared between groups using Kruskal–Wallis rank sum tests. If no significant differences were observed between the two groups of MSM, their data were combined. Pathogenic and non-pathogenic *Neisseria* were described and analysed separately, and subsequently stratified by species for species that were isolated at least once in each group.

In a sensitivity analysis, we used linear regression with geometric mean MIC as the outcome and two binary dependent variables: (a) being MSM/employee, and (b) antimicrobial exposure in the previous 6 months. The model was also adjusted for *Neisseria* species by the inclusion of a categorical variable.

All statistical analyses were performed with R version 4.0.5 (R Foundation for Statistical Computing, Vienna, Austria).

### Ethics

Ethics approval was obtained from ITM’s Institutional Review Board (1276/18 and 1351/20) and from the Ethics Committee of the University of Antwerp (19/06/058 and AB/ac/003).

The study was carried out according to the principles stated in the Declaration of Helsinki, all applicable regulations and according to the most recent GCP and GCLP guidelines. The Informed Consent Form (ICF) documents were designed in accordance with the requirements of the Helsinki Declaration (2013), the E6 ICH GCP Guidelines (2016) and the Belgian Law on Experiment on the Human Person (2004).

## Results

The median age of the 96 participants was 35 (IQR 35–47.5) years (Table 1). Among the employees, two thirds were female. The MSM reported a high rate of partner change and a low rate of condom use, which is compatible with the high incidence of sexually transmitted infections in the PReGo study^27^. Of the 32 MSM who used antimicrobials in the previous 6 months, 14 (43.8%) used only one class of antimicrobials, 14 (43.8%) used two different classes of antimicrobials, and four (12.5%) participants used three different classes of antimicrobials Supplementary information.

**Table 1:** Population characteristics.

	Overall (n = 96)	Employees (n = 32)	MSM who did not use antibiotics (n = 32)	MSM who used antibiotics (n = 32)	*p-*value*
Age in years, median (IQR)	35 (35–47.5)	45 (35–55)	45 (35–55)	39 (35–45)	0.21
Male sex, n (%)	74 (77.1)	10 (31.3)	32 (100.0)	32 (100.0)	< 0.001
**Antibiotic exposure in the previous 6 months, n (%)**	32 (33.3)	0 (0.0)	0 (0.0)	32 (100.0)	NA
β-Lactams	25 (26.0)	NA	NA	25 (78.1)	NA
Macrolides	19 (19.8)			19 (59.4)	
Fluoroquinolones	2 (2.1)			2 (6.3)	
Other	8 (8.3)			8 (25.0)	
**Antibiotic exposure in the previous 1 month, n (%)**	7 (7.3)	0 (0.0)	0 (0.0)	7 (21.9)	NA
β-Lactams	4 (4.2)	NA	NA	4 (12.5)	NA
Macrolides	0 (0.0)			0 (0.0)	
Fluoroquinolones	1 (1.0)			1 (14.3)	
Other	2 (2.1)			2 (6.3)	
Median number of casual sex partners in the previous 3 months	NA	NA	10.0 (4.8–15.0)	10.0 (8.0–20.0)	0.12
Used condoms with > 75% of casual anal sex partners in the previous 3 months, n (%)	NA	NA	9 (28.1)	2 (6.5)^a^	0.03
Used a mouthwash in the previous 1 month, n (%)	46 (47.9)	15 (46.9)	12 (37.5)	19 (59.4)	0.22

*NA* not applicable/not available.

*Kruskal–Wallis rank sum test.

^a^1 missing value.

### *Neisseria* prevalence

**Figure 1:**
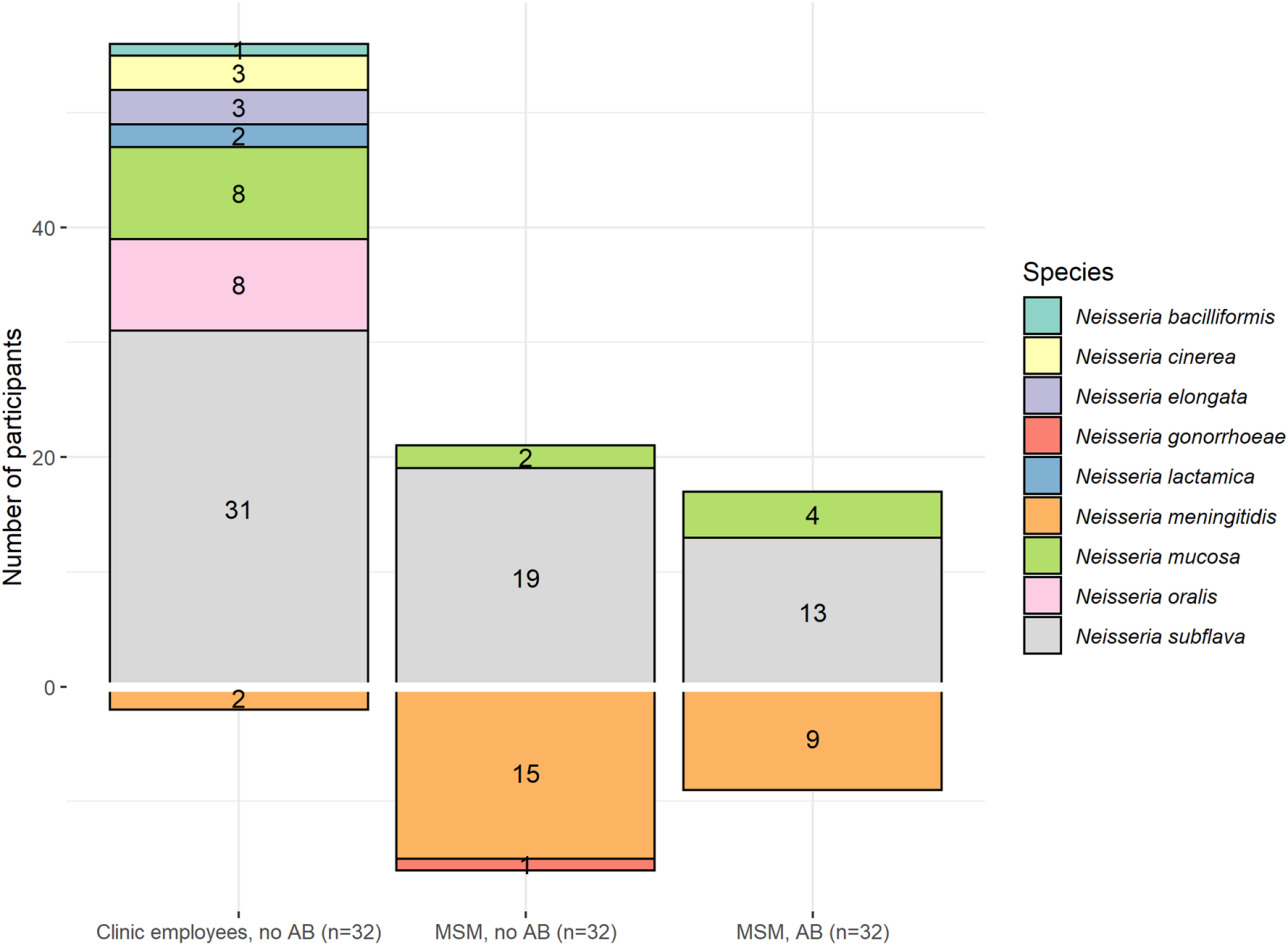
Prevalence and richness of *Neisseria* species, in absolute number of participants from whom the concerning species was isolated, per group. *AB* antibiotics, *MSM* men who have sex with men.

In total 207 *Neisseria* colonies were isolated, representing seven non-pathogenic and two pathogenic species (Table 2, Fig. 1). In descending order of prevalence, we isolated the non-pathogenic species *N. subflava* (63/96, 65.6%), *N. mucosa* (14/96, 14.6%)*, N. oralis* (8/96, 8.3%), *N. cinerea* (3/96, 3.1%)*, N. elongata* (3/96, 3.1%)*, N. lactamica* (2/96, 2.1%), and *N. bacilliformis* (1/96, 1.0%). The pathogenic species were *N. meningitidis* (26/96, 27.1% prevalence), and *N. gonorrhoeae* (one isolate from a MSM, 1.0% prevalence).

**Table 2:** Antimicrobial susceptibility of *Neisseria* isolates cultured from the oropharynx of 64 STI clinic attendees (men who have sex with men) and 32 employees of the Institute of Tropical Medicine (representing the general population) in Belgium.

	Prevalence (n/N) Participants (%)	Azithromycin (mg/L) Median (IQR)	Ciprofloxacin (mg/L) Median (IQR)	Ceftriaxone (mg/L) Median (IQR)
**Pathogenic *Neisseria* spp.**	27/96 (28.1)	0.5 (0.4–0.9)	0.004 (0.003–0.006)	< 0.016 (< 0.016–< 0.016)
*Neisseria meningitidis*	26/96 (27.1)	0.5 (0.3–0.9)	0.004 (0.003–0.005)	< 0.016 (< 0.016–< 0.016)
Employees	2/32 (6.3)	1.0 (0.8–1.3)	0.065 (0.034–0.095)	< 0.016 (< 0.016–< 0.016)
MSM who used AB previous 6 months	9/32 (28.1)	0.8 (0.5–1.5)	0.004 (0.002–0.006)	< 0.016 (< 0.016–0.012)
MSM who used no AB previous 6 months	15/32 (46.9)	0.5 (0.4–0.5)	0.004 (0.003–0.004)	< 0.016 (< 0.016–< 0.016)
*Neisseria gonorrhoeae*	1/96 (1.0)	0.125	2.0	< 0.016
Employees	0/32 (0.0)	–	–	–
MSM who used AB previous 6 months	0/32 (0.0)	–	–	–
MSM who used no AB previous 6 months	1/32 (3.1)	0.125	2.0	< 0.016
**Non-pathogenic *Neisseria* spp.**	65/96 (67.7)	3.0 (2.0–7.5)	0.032 (0.016–0.25)	0.047 (0.029–0.064)
Employees	32/32 (100.0)	3.0 (2.0–4.0)	0.023 (0.012–0.064)	0.034 (0.026–0.064)
MSM who used AB previous 6 months	19/32 (59.4)	16.0 (3.0–> 256.0)	0.250 (0.141–0.500)	0.047 (0.032–0.094)
MSM who used no AB previous 6 months	14/32 (43.8)	4.0 (3.0–48.0)	0.125 (0.016–0.380)	0.047 (0.032–0.064)
*Neisseria subflava*	63/96 (65.6)	3.5 (2.5–16.0)	0.125 (0.016–0.380)	0.047 (0.028–0.064)
Employees	31/32 (96.9)	3.0 (2.3–4.0)	0.032 (0.016–0.197)	0.035 (0.028–0.052)
MSM who used AB previous 6 months	13/32 (40.6)	288 (3.5–> 256.0)	0.380 (0.190–0.500)	0.064 (0.032–0.064)
MSM who used no AB previous 6 months	19/32 (59.4)	4.0 (3.3–72.0)	0.125 (0.022–0.380)	0.047 (0.028–0.126)
*Neisseria mucosa*	14/96 (14.6)	3.5 (2.3–5.5)	0.016 (0.013–0.030)	0.040 (0.032–0.064)
Employees	8/32 (25.0)	3.5 (2.8–4.5)	0.017 (0.011–0.025)	0.040 (0.032–0.072)
MSM who used AB previous 6 months	4/32 (12.5)	3.5 (2.8–6.3)	0.133 (0.015–1.688)	0.040 (0.032–0.051)
MSM who used no AB previous 6 months	2/32 (6.3)	12.6 (6.9–18.3)	0.016 (0.016–0.016)	0.063 (0.048–0.079)
*Neisseria oralis*	8/96 (8.3)	2.0 (1.9–3.1)	0.015 (0.012–0.018)	0.056 (0.032–0.064)
Employees	8/32 (25.0)	2.0 (1.0–3.1)	0.015 (0.012–0.018)	0.056 (0.032–0.064)
MSM who used AB previous 6 months	0/32 (0.0)	–	–	–
MSM who used no AB previous 6 months	0/32 (0.0)	–	–	–
*Neisseria cinerea*	3/96 (3.1)	2.0 (1.5–15.0)	0.012 (0.009–0.022)	< 0.016 (< 0.016–< 0.016)
Employees	3/32 (9.4)	2.0 (1.5–15.0)	0.012 (0.009–0.022)	< 0.016 (< 0.016–< 0.016)
MSM who used AB previous 6 months	0/32 (0.0)	–	–	–
MSM who used no AB previous 6 months	0/32 (0.0)	–	–	–
*Neisseria elongata*	3/96 (3.1)	0.5 (0.4–0.6)	0.004 (0.004–0.014)	0.047 (0.035–0.119)
Employees	3/32 (9.4)	0.5 (0.4–0.6)	0.004 (0.004–0.014)	0.047 (0.035–0.119)
MSM who used AB previous 6 months	0/32 (0.0)	–	–	–
MSM who used no AB previous 6 months	0/32 (0.0)	–	–	–
*Neisseria lactamica*	2/96 (2.1)	1.5 (1.3–1.8)	0.127 (0.096–0.159)	< 0.016 (< 0.016–< 0.016)
Employees	2/32 (6.3)	1.5 (1.3–1.8)	0.127 (0.096–0.159)	< 0.016 (< 0.016–< 0.016)
MSM who used AB previous 6 months	0/32 (0.0)	–	–	–
MSM who used no AB previous 6 months	0/32 (0.0)	–	–	–
*Neisseria bacilliformis*	1/96 (1.0)	2 (–)	0.125 (–)	1.5 (–)
Employees	1/32 (3.1)	2 (–)	0.125 (–)	1.5 (–)
MSM who used AB previous 6 months	0/32 (0.0)	–	–	–
MSM who used no AB previous 6 months	0/32 (0.0)	–	–	–

*AB* antibiotics, *IQR* interquartile range, *MSM* men who have sex with men, *STI* sexually transmitted infections.

The prevalence of non-pathogenic *Neisseria* was lower among MSM (51.6%) than the employees (100.0%, *p* < 0.00001, Table 2, Fig. 1), but for the pathogenic *Neisseria* this was the reverse: *N. meningitidis* was much more prevalent among MSM (37.5%) than the employees (6.3%, *p* < 0.01).

MSM who used antimicrobials in the previous 6 months were less often colonised with *N. meningitidis* (28.1%) than MSM who did not use antibiotics (46.9%), but this difference was not statistically significant (*p* = 0.20).

### Richness of non-pathogenic *Neisseria* species

Co-colonisation with multiple non-pathogenic *Neisseria* species was less common among MSM (7.8% were colonised with two species) than the employees (37.5% colonised with two species and 18.8% with three species).

In addition, while all seven non-pathogenic species were isolated from the employees, only two were isolated from MSM: *N. subflava* and *N. mucosa*. The richness of non-pathogenic species was thus lower among MSM (median of 1 species, IQR 0–1) than the employees (median of 2 species, IQR 1–2, *p* < 0.0001).

### Susceptibility of non-pathogenic *Neisseria*

**Figure 2:**
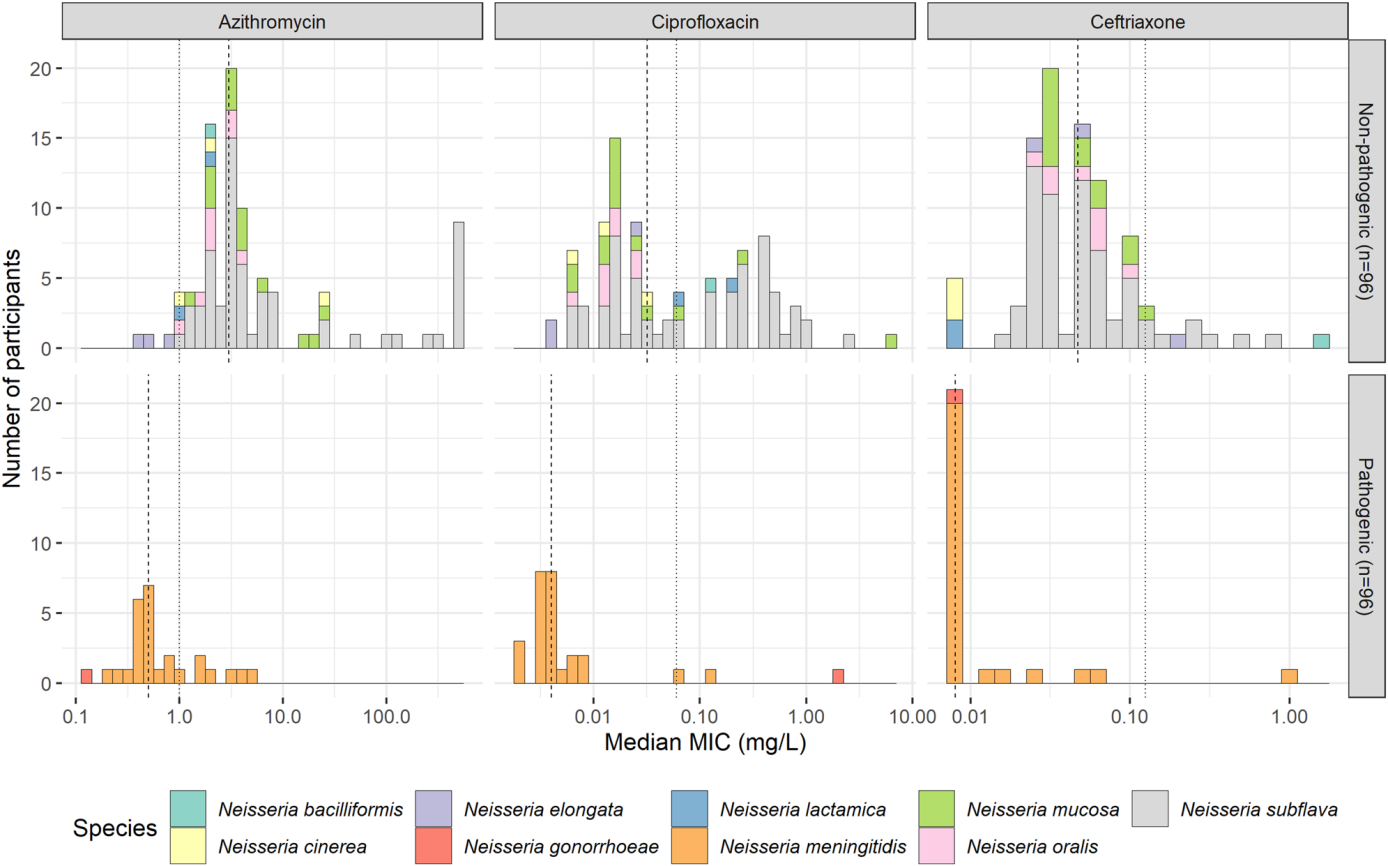
Minimum inhibitory concentration (MIC, mg/L) of pathogenic versus non-pathogenic *Neisseria* species isolated from all 96 participants. Numbers represent the number of participants with that specific median MIC per species. Vertical lines indicate the median of median MICs (dashed line) and the EUCAST v.11.0 cutoff for *N. gonorrhoeae* (dotted line) for each antibiotic.

**Figure 3:**
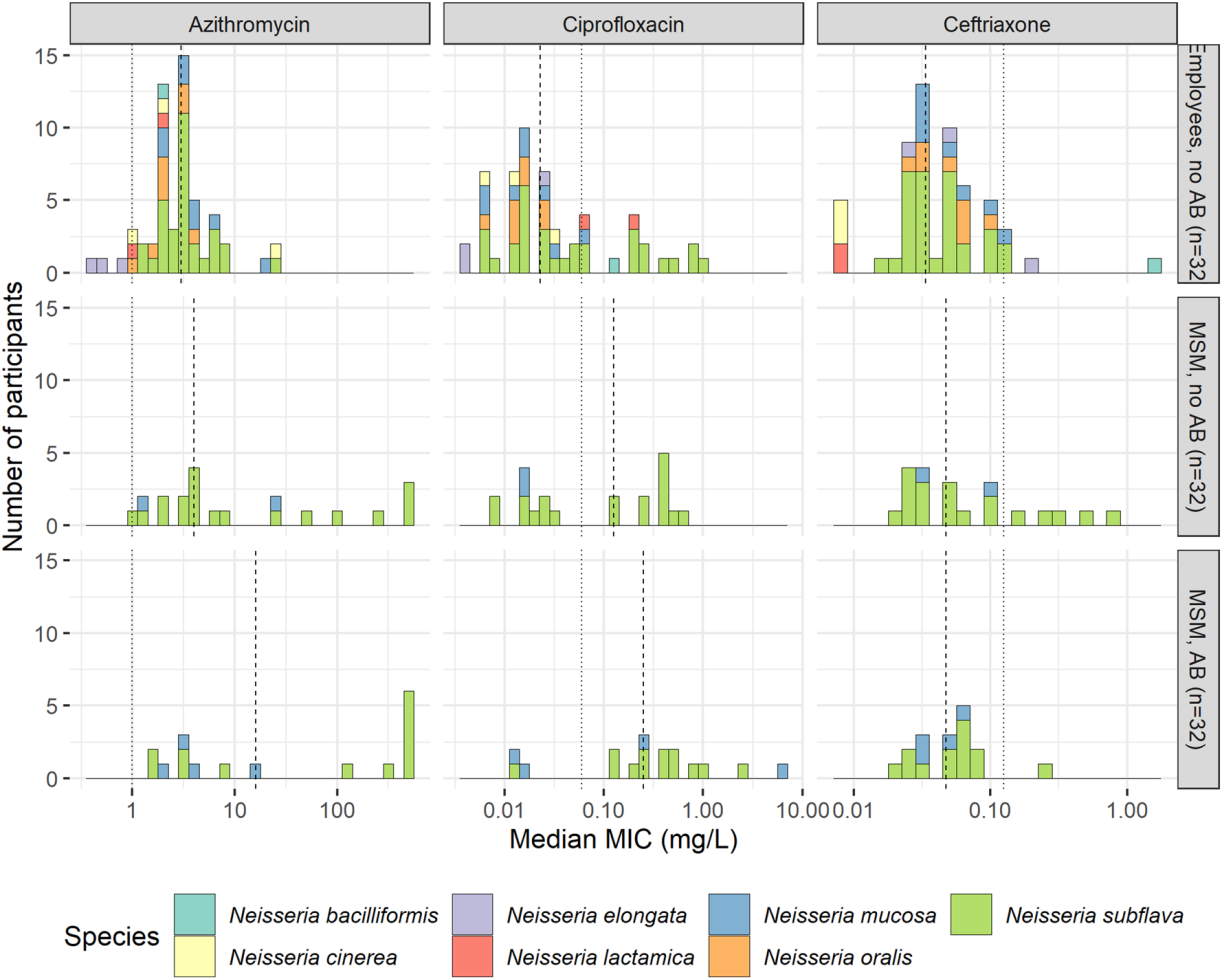
Minimum inhibitory concentration (MIC, mg/L) of non-pathogenic *Neisseria* species, per group. Numbers represent the number of participants with that specific median MIC per species. Vertical lines indicate the median of median MICs (dashed line) and the EUCAST v.11.0 cutoff for *N. gonorrhoeae* (dotted line) for each antibiotic.

The non-pathogenic *Neisseria* were significantly less susceptible (higher MICs) to all three antimicrobials than the pathogenic *Neisseria* (*p* < 0.0001 for every antimicrobial, Table 2, Fig. 2). The non-pathogenic *Neisseria* isolated from MSM had significantly higher MICs for azithromycin (7.0 mg/L, IQR 3.0–280.2) and ciprofloxacin (0.250 mg/L, IQR 0.020–0.380) compared to those from the employees (3.0 mg/L, IQR 2.0–4.0, *p* < 0.0001; and 0.023 mg/L, IQR 0.012–0.064, *p* < 0.001, respectively; Table 2, Fig. 3). The MICs for ceftriaxone were similar in both groups (0.047 mg/L, IQR 0.032–0.084 in MSM versus 0.034, IQR 0.026–0.064 in the employees, *p* = 0.3). There were no significant differences in MICs according to recent antimicrobial exposure in MSM. The stratified analysis for *N. subflava* showed similar findings. The stratified analysis for *N. mucosa* showed no significant differences in MICs between groups.

The sensitivity analysis based on a linear regression model confirmed the association between MSM and higher MICs for azithromycin (aOR 3.31, 95% CI 1.42–7.72), but estimated an additional increase with recent antimicrobial use (aOR 2.99, 95% CI 1.07–8.31).

For ciprofloxacin, the model suggested that the difference in MIC is only driven by higher MICs in those who were recently exposed to antimicrobials (aOR 3.79, 95% CI 1.49–9.59, Table 3). In addition, the model estimated an association between MSM and higher MICs for ceftriaxone (aOR 1.58, 95% CI 1.06–2.35).

**Table 3:** Linear regression coefficients for change in geometric mean minimum inhibitory concentrations of non-pathogenic *Neisseria* for ciprofloxacin, azithromycin and ceftriaxone.

All non-pathogenic *Neisseria*	Number of participants (%)	Ciprofloxacin		Azithromycin		Ceftriaxone	
		Unadjusted OR (95% CI)	Adjusted^A^ OR (95% CI)	Unadjusted OR (95% CI)	Adjusted^A^ OR (95% CI)	Unadjusted OR (95% CI)	Adjusted^A^ OR (95% CI)
**Population**							
Employees	32 (33.3)	1 (Ref)	1 (Ref)	1 (Ref)	1 (Ref)	1 (Ref)	1 (Ref)
MSM	64 (66.7)	2.45 (1.14–5.27)*	1.69 (0.78–3.66)	4.38 (1.97–9.77)*	3.31 (1.42–7.72)*	1.66 (1.05–2.61)*	1.58 (1.06–2.35)*
**Used antibiotic in the previous 6 months**							
No	64 (66.7)	1 (Ref)	1 (Ref)	1 (Ref)	1 (Ref)	1 (Ref)	1 (Ref)
Yes	32 (33.3)	3.23 (1.21–8.59)*	3.79 (1.49–9.59)*	2.69 (0.97–7.47)	2.99 (1.07–8.31)*	0.75 (0.42–1.34)	0.75 (0.47–1.21)

*Neisseria subflava*	Number of participants (%)	Unadjusted	Adjusted^A^	Unadjusted	Adjusted^A^	Unadjusted	Adjusted^A^
**Population**							
Employees	31 (49.2)	1 (Ref)	NA	1 (Ref)	NA	1 (Ref)	NA
MSM	32 (50.8)	1.80 (0.75–4.33)	NA	4.07 (1.51–10.95)*	NA	1.68 (1.06–2.67)*	NA
**Used antibiotic in the previous 6 months**							
No	50 (79.4)	1 (Ref)	NA	1 (Ref)	NA	1 (Ref)	NA
Yes	13 (20.6)	3.34 (1.13–9.86)*	NA	4.58 (1.35–15.57)*	NA	0.78 (0.44–1.38)	NA

*CI* Confidence Interval, *MIC* minimum inhibitory concentration, *NA* not applicable, *OR* odds ratio.

*Estimate is statistically significant as the CI does not include 1.

^A^Adjusted for *Neisseria* species.

### Susceptibility of pathogenic *Neisseria*

For *N. meningitidis*, most isolates were highly susceptible to all three antimicrobials. According to current EUCAST breakpoints (v. 11.0), one isolate was resistant to ceftriaxone (MIC 1 mg/L) and two participants had isolates with ciprofloxacin resistance (MIC 0.125 and 0.064 mg/L).

The single *N. gonorrhoeae* isolate in this survey was susceptible to azithromycin (MIC 0.125 mg/L) and ceftriaxone (MIC < 0.016 mg/L) but resistant to ciprofloxacin (MIC 2 mg/L).

## Discussion

We found that contemporary oropharyngeal non-pathogenic *Neisseria* in MSM were less susceptible to antimicrobials than those from employees representing the general population. Recent antimicrobial exposure did not entirely explain the observed differences in susceptibility. This suggests that long-term participant- or population-level antimicrobial exposure plays an important role^29^. Indeed, MSM in PrEP programs consume a large amount of antimicrobials. One of the main drivers of excessive macrolide and cephalosporin consumption among PrEP users is the practice of screening asymptomatic MSM for gonorrhoea and chlamydia^30^. In some cohorts, macrolide consumption exceeds 12 defined daily doses per 1000 individuals per day (DID)^30^. This is multiple times what is consumed by typical general populations and is above the thresholds for inducing macrolide resistance in a range of bacterial species^30,31^. Reducing the intensity of screening for gonorrhoea and chlamydia among MSM may result in a four-fold decrease in macrolide consumption^32^.

Although lower than in MSM, the MICs of non-pathogenic *Neisseria* in the employees were considerably higher than in previous surveys. This is illustrated by *N. subflava*, the most prevalent species in our survey. A previous analysis of *N. subflava* isolates from the early 1980s found a considerably lower azithromycin MIC distribution (median 1.0 mg/L, IQR 0.5–2.5 mg/L) than that found in the current employees (median 3.0 mg/L, IQR 2.3–4.0 mg/L)^16^.

In fact, the antimicrobial susceptibilities of the non-pathogenic *Neisseria* from the employees in our study were all higher than those from published reports from equivalent studies in the 1960s to the 1990s^33–37^. Of note, the earliest survey of antimicrobial susceptibility in commensal *Neisseria* that we could locate, found that 28 clinical isolates of *N. cinerea* from Germany pre-1961 were highly susceptible to penicillin (MIC range 0.00015–0.0006 mg/L)^33^. A likely explanation for this decrease in antimicrobial susceptibility over time is the level of antimicrobial consumption by the general Belgian population^38^. Macrolide consumption, for example, exceeded 3.0 DID in 2018 and 2019, which is well above a threshold of 1.3 DID which may select for resistance in pathogens like *S. pneumoniae, M. genitalium,* and *T. pallidum*^31,39^. Certain features of commensal bacteria suggest that such resistance threshold may even be lower for commensals than for pathogens. Thus, population-level antimicrobial consumption may have selected for circulating commensal *Neisseria* with elevated MICs (“Supplementary information”).

The prevalence and richness of non-pathogenic *Neisseria* among MSM in our survey was lower than the employees and much lower than reported among MSM in Vietnam and the USA^8,40^. These low numbers among Belgian MSM taking PrEP could be explained by the high antimicrobial exposure of this population^30^. Similar to *N. meningitidis*, certain species of non-pathogenic *Neisseria* may be slower to acquire resistance to specific antimicrobials than other species^9,13^. For example, no isolates of *N. elongata, N. lactamica* or *N. bacilliformis* in our study had an azithromycin MIC greater than 2 mg/L, whereas the median azithromycin MIC for *N. subflava* was 3 mg/L in the employees, 8 mg/L in MSM overall and 288 mg/L in the MSM group that had used antibiotics. This high-level resistance to azithromycin in *N. subflava* has been linked to the uptake of an *msrD* gene likely from oral streptococci^41^. Other *Neisseria* species have thus far not been found to be able to take up this gene or acquire such high-level resistance to azithromycin^41^. The higher consumption of antimicrobials in this MSM PrEP cohort could thus have eliminated the most susceptible non-pathogenic *Neisseria* species and thereby have reduced *Neisseria* species richness.

Conversely, the prevalence of *N. meningitidis* in our study was higher among MSM than the employees, which corroborates other reports of *N. meningitidis* prevalences up to 42.5% among MSM^21–24^. This exceeds by some margin the prevalence in young adults across the globe^42^. *N. meningitidis* is one of the most antimicrobial susceptible *Neisseria* species, as also observed in our current study^43^. A number of genetic differences between *N. meningitidis* and other *Neisseria* have been shown to underpin the reduced capacity of *N. meningitidis* to acquire resistance to various antimicrobials^44,45^.

Indeed, in our study, the prevalence of *N. meningitidis* in MSM exposed to antimicrobials was almost half that in unexposed MSM. The prevalence of *N. meningitidis* may thus temporarily decline due to the consumption of antimicrobials (as also shown in other studies^21^), but soon return to its equilibrium prevalence.

Several processes could explain the higher prevalence of *N. meningitidis* among MSM compared with members of the general population. One reason may be the high frequency of interpersonal contacts among MSM taking PrEP—like kissing and attending crowded night-clubs—during which transmission may occur^21,46^. Hypothetically, *N. meningitidis* may be more transmissible than non-pathogenic *Neisseria* and may thus outcompete the latter in recolonizing the pharynx after antimicrobial exposure. Lack of competition with other *Neisseria* species may be another explanation. A number of epidemiological, interventional and in-vitro studies have found evidence of such competition^47^. As an example, the presence of *N. lactamica* has been shown to be associated with a lower prevalence of *N. meningitidis*^48–50^.

If antibiotics reduced the prevalence of species such as *N. lactamica* in MSM, this may have left this population more susceptible to colonisation by *N. meningitidis.*

This study has a number of limitations, including the small sample sizes, single centre design and the fact that the samples were not representative of all MSM or the general Belgian population. Furthermore, two experimental factors of this survey may have caused underestimation of the richness of *Neisseria* species and the spectrum of their antibiotic susceptibilities. Firstly, the study depended on culturing *Neisseria* from the posterior oropharynx and tonsils. This design would likely have missed certain non-pathogenic *Neisseria* that preferentially inhabit other parts of the pharynx^51^. Future studies could obtain samples by gargling with physiological saline to overcome this problem^51^. Secondly, only a minority of colonies grown on the agar plates were selected for species identification and MIC determination. We tried to pick at least one of each macroscopically distinct gram negative and oxidase positive colony per plate, but we may have missed particular *Neisseria* species with phenotypes similar to the sampled colonies. Metagenomic studies may also be a more sensitive way to profile the *Neisseria* microbiota and resistome than culture-based techniques. Finally, it would be instructive to repeat this study in settings with low population level antibiotic consumption.

In conclusion, we found high levels of resistance to azithromycin, ceftriaxone, and ciprofloxacin in oropharyngeal *Neisseria* among MSM and employees in Belgium. This finding is worrisome as non-pathogenic *Neisseria* provide a reservoir of resistance genes that can be readily transferred to pathogenic bacteria.

This AMR is most parsimoniously explained by excessive antibiotic exposure in the general Belgian population, but particularly in the MSM PrEP cohorts. Reduced screening for asymptomatic gonorrhoea and chlamydia may substantially reduce antimicrobial consumption by MSM.

The effect of such a policy change on the prevalence of AMR may be most easily demonstrated in the non-pathogenic *Neisseria*. Future studies may thus consider conducting regular surveys of antimicrobial susceptibility of non-pathogenic *Neisseria* in the general population and key populations such as MSM on PrEP as an early warning system of excessive antimicrobial consumption.

## Supplementary Information

Supplementary Information.

## Data Availability

All deidentified data are available as a Supplement to this manuscript. Additional related documents such as the study protocol, laboratory analysis plan, informed consent form can be obtained from the corresponding author upon reasonable request.
